# Supplementary material for: Development of Charge-Augmented Three-Point Water Model (CAIPi3P) for Accurate Simulations of Intrinsically Disordered Proteins
Source: Int J Mol Sci. 2020 Aug 26;21(17):6166. doi: 10.3390/ijms21176166 (PMC7504337; doi:10.3390/ijms21176166)
Supplement: Supplementary file 1 [file ijms-21-06166-s001.pdf]

International Journal of *Molecular Sciences*

MDPI

Supplementary Materials

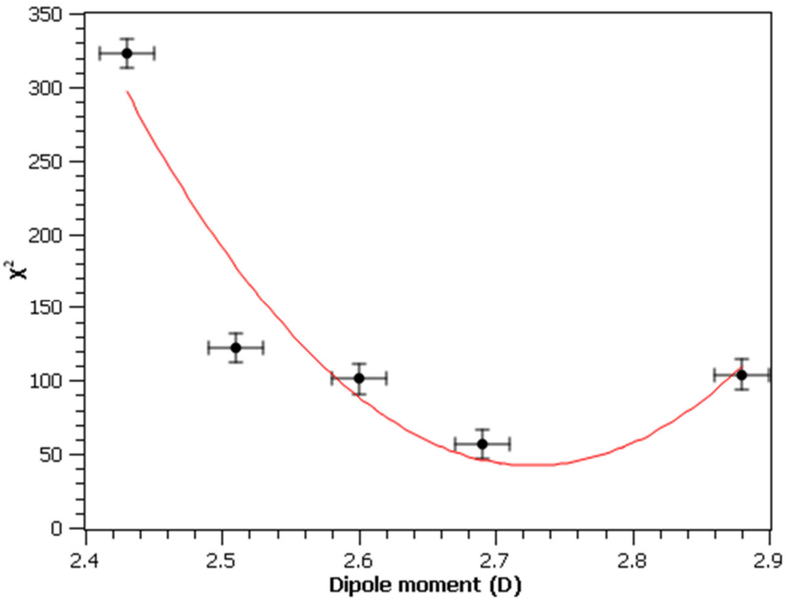

Figure S1. Optimization Curve for different tested water dipole moment values in comparison to the χ2 values for Histatin5.

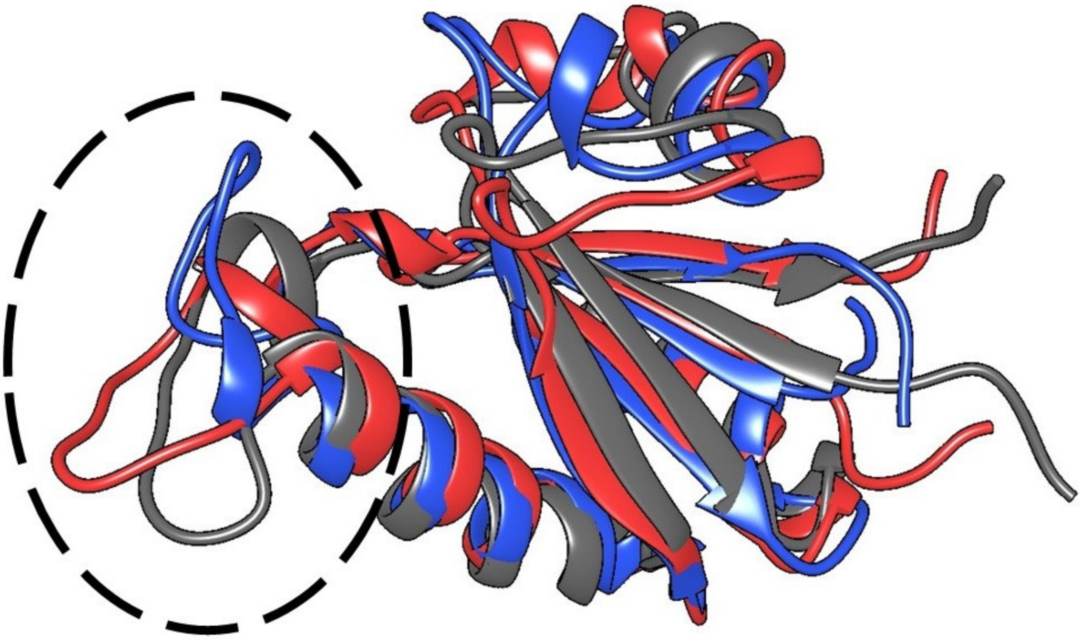

Figure S2. Average structures obtained from different simulations of the LaRP-RRM1 – Blue: AMBER99SB+TIP3P. Red: AMBER03ws+CAIPi3P, Grey : average structure from the NMR ensemble (PDB: 2MTG). Inside the dashed lines is the loop A199-G206.

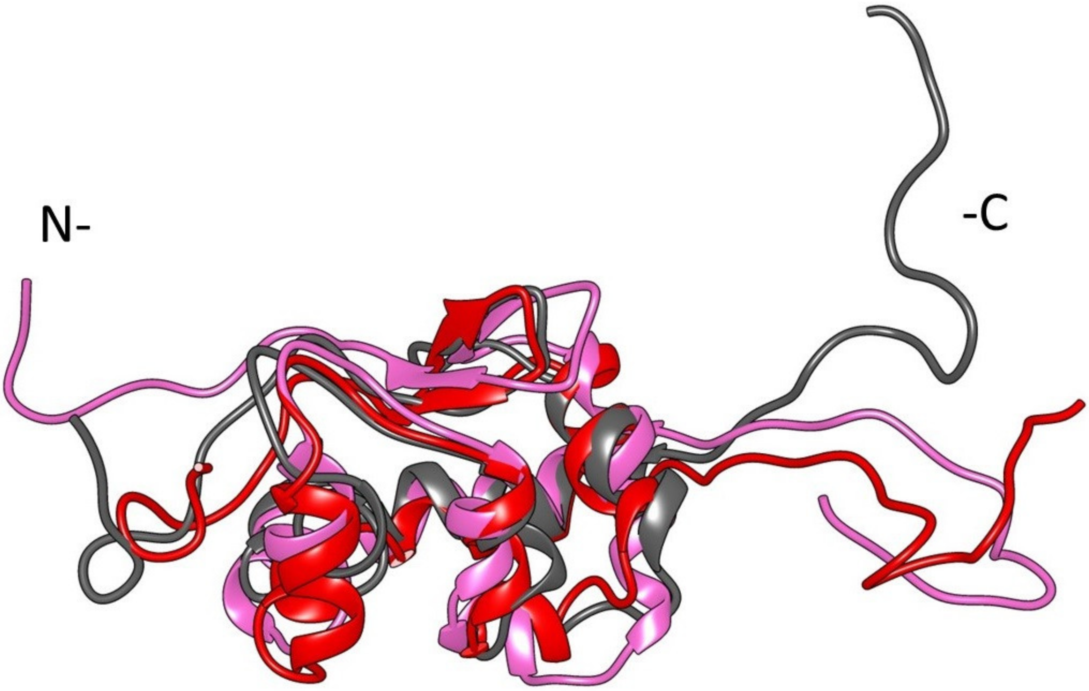

Figure S1. Average structures obtained from different simulations of the LaRP-LaM. Pink – AMBER03ws+TIP4P/2005. Red: AMBER03ws+CAIPi3P, Grey: average structure from the NRM ensemble (PDB: 2MTF).

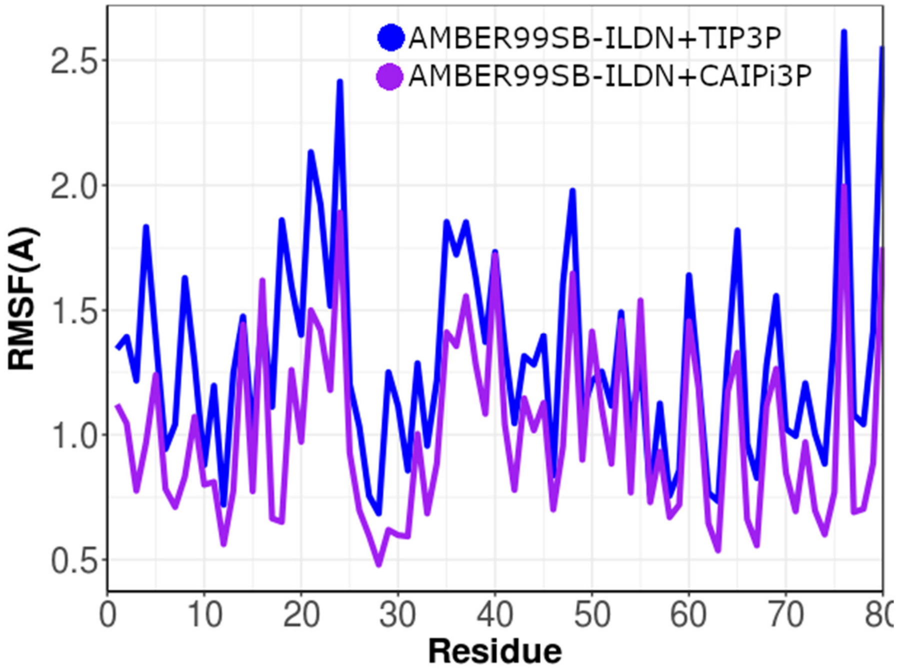

Figure S4. Lysozyme RMSF per residue.

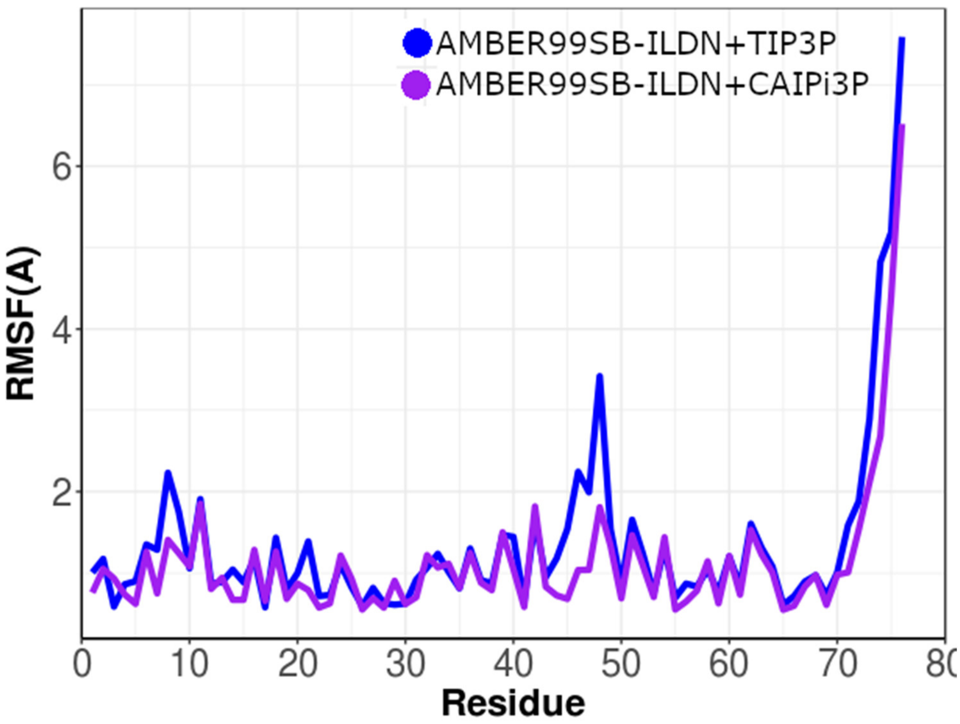

Figure S5. Ubiquitin RMSF per residue.

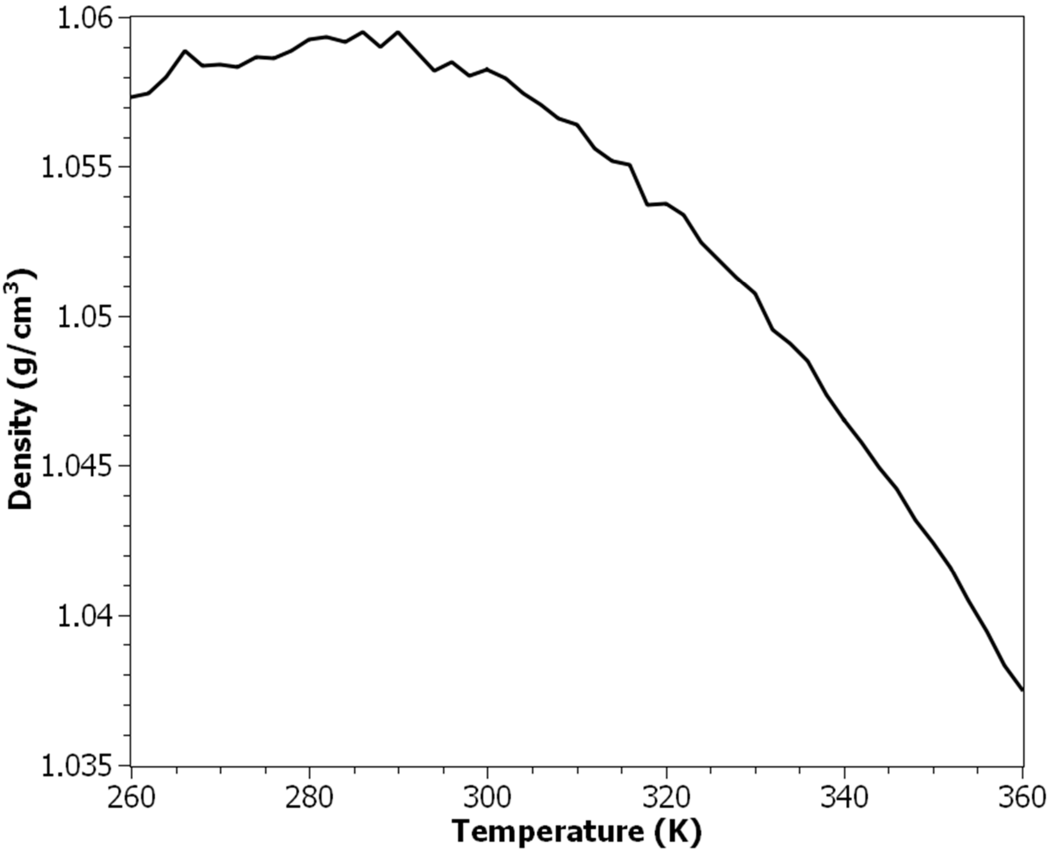

Figure S6. Density in function of temperature. The maximum density is located in 286K.

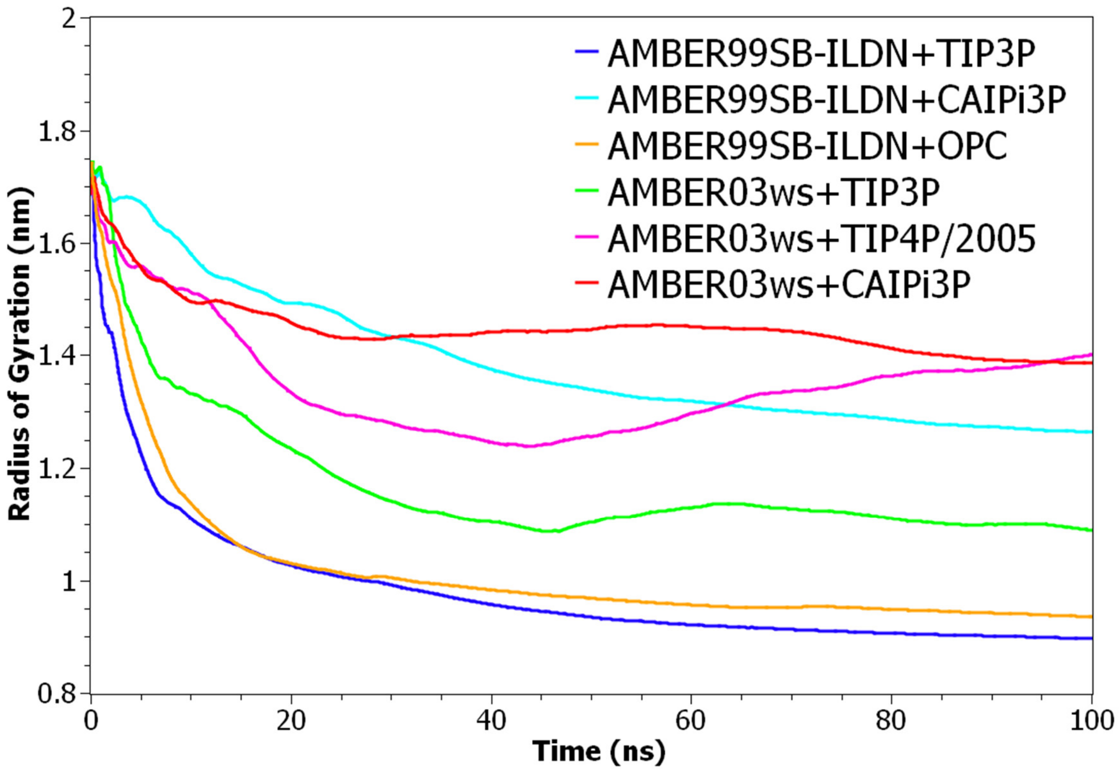

Figure S7. Radius of gyration convergence for different combinations of Force-Fields and water models for the Histatin5.

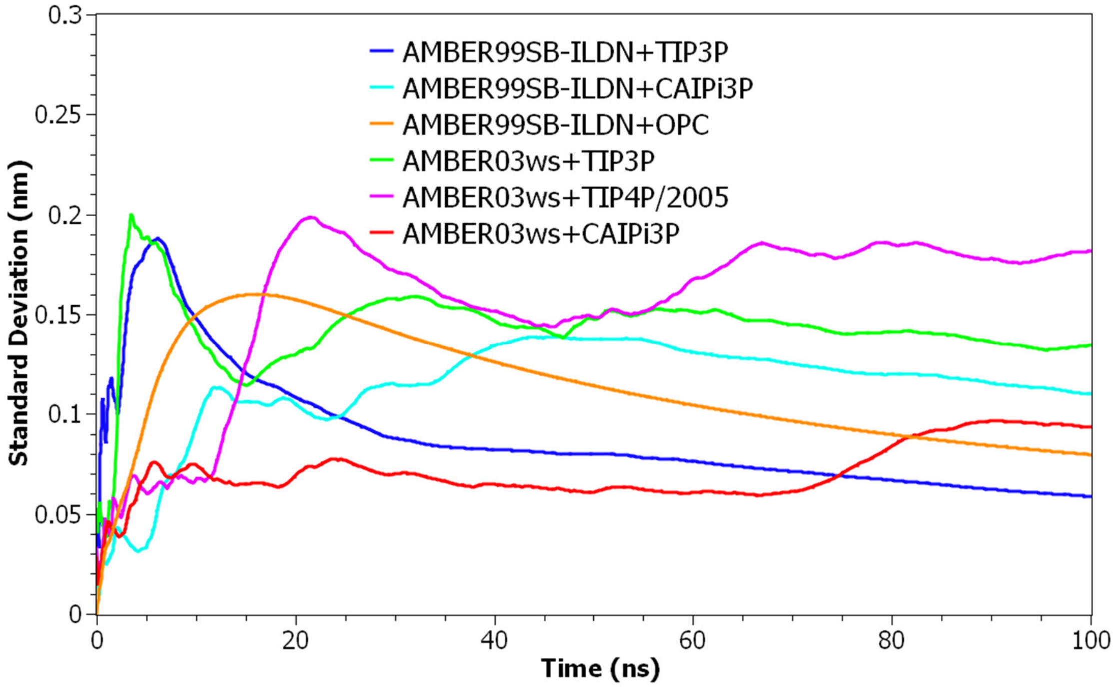

Figure S8. Radius of gyration convergence for different combinations of Force-Fields and water models for the Histatin5.

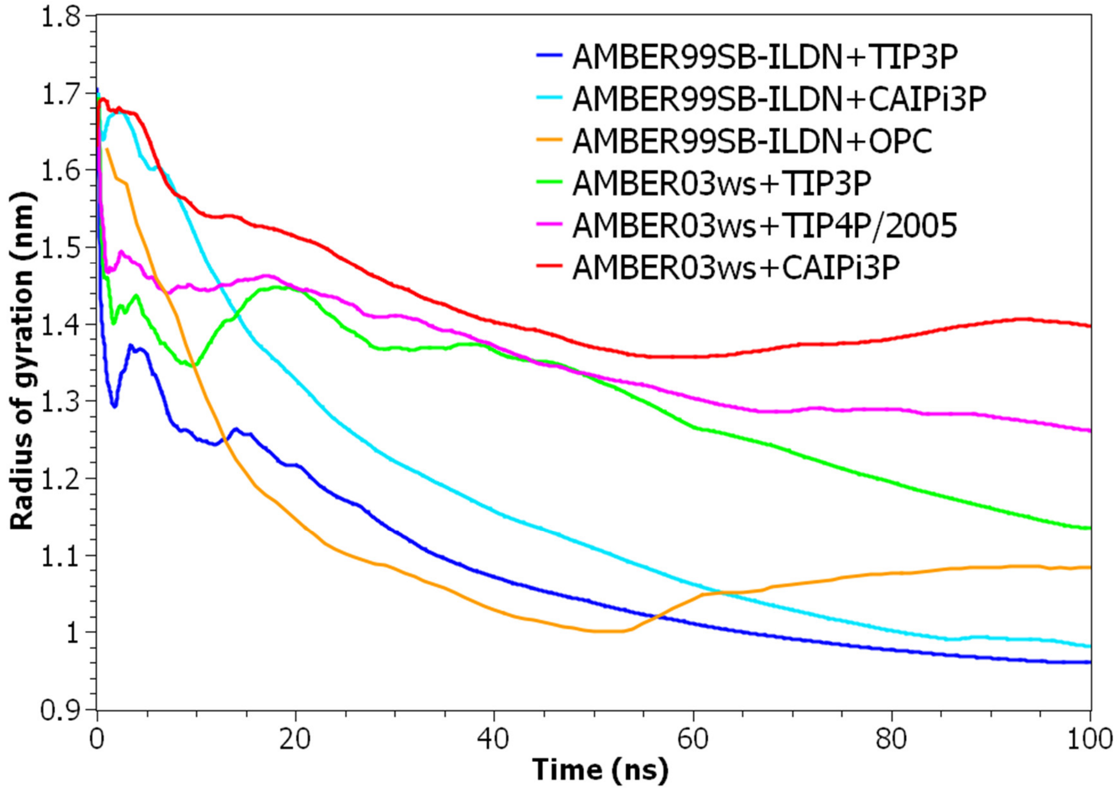

Figure S9. Radius of gyration convergence for different combinations of Force-Fields and water models for the RS-pep.

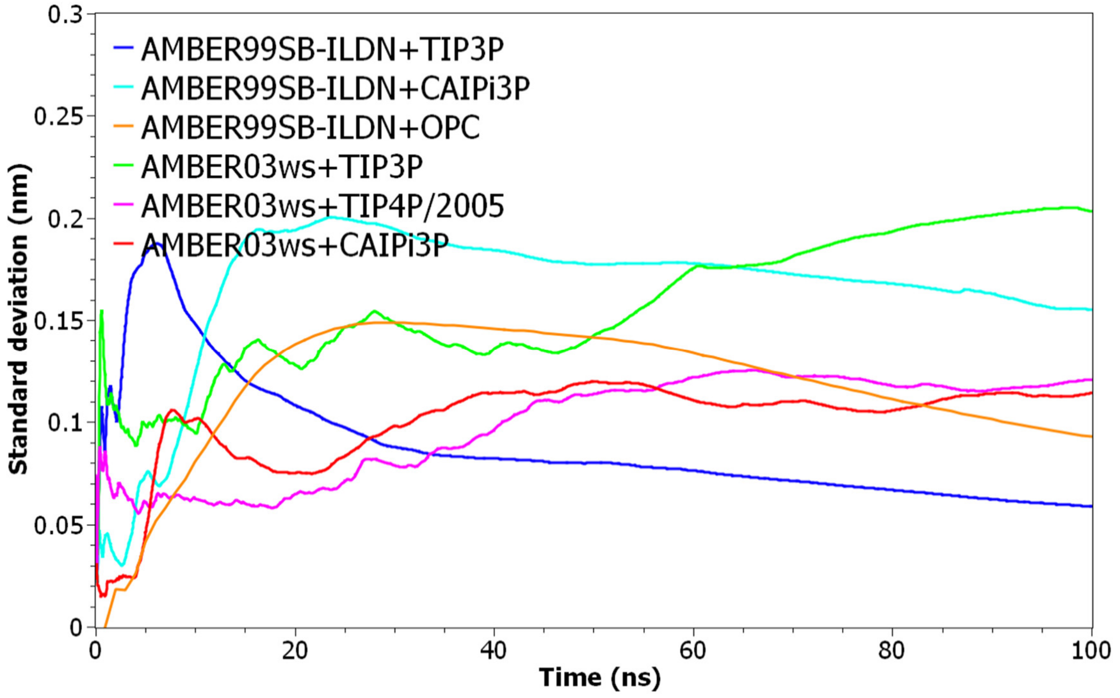

Figure S10. Average radius of gyration standard deviation convergence for different combinations of Force-Fields and water models for the RS-pep.

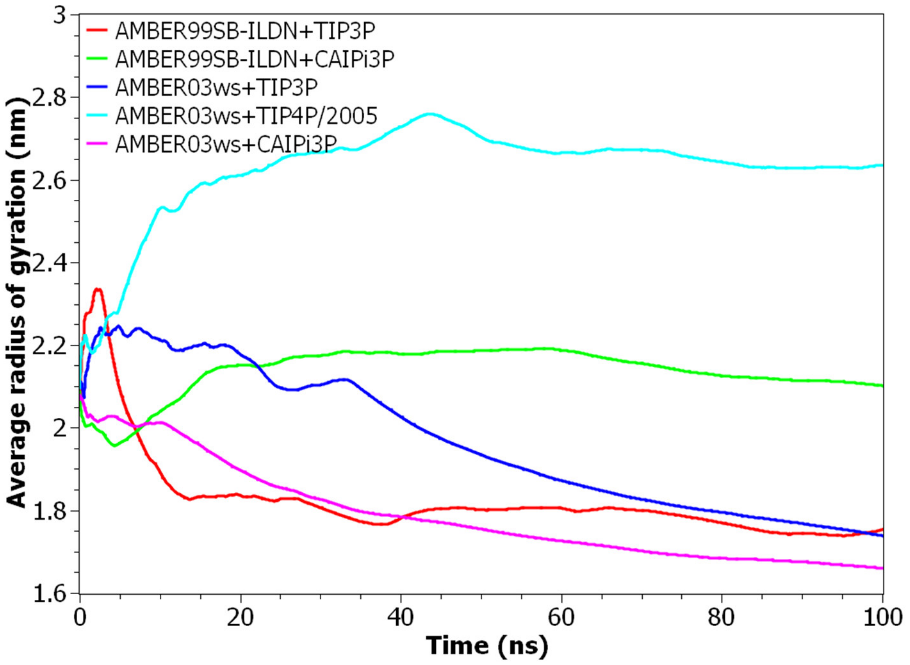

Figure S11. Radius of gyration convergence for different combinations of Force-Fields and water models for the At2g23090.

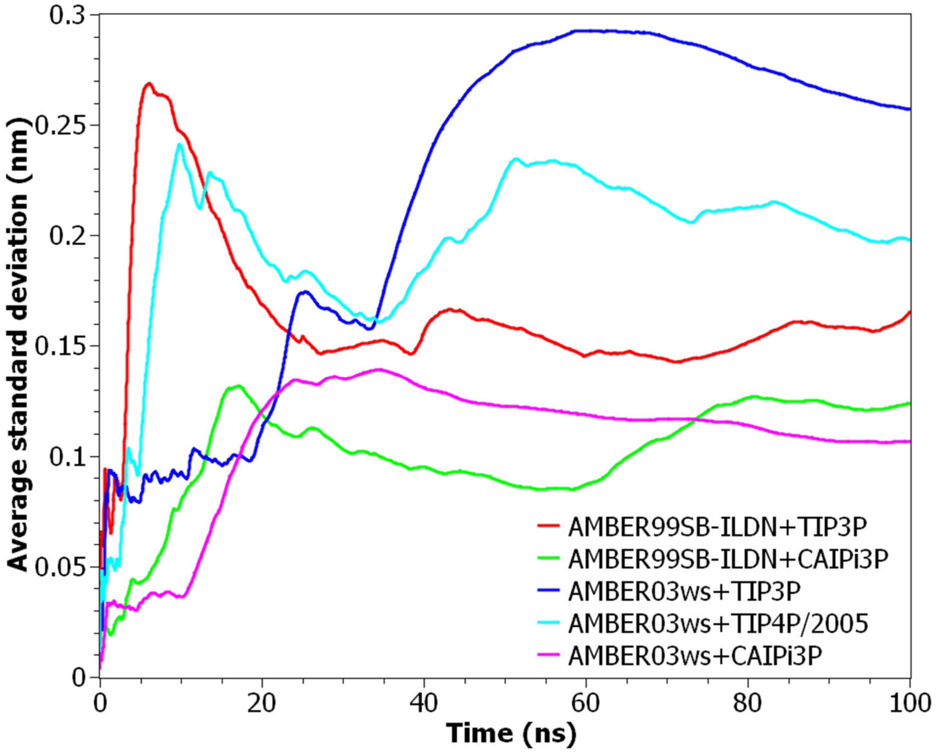

Figure S12. Radius of gyration standard deviation convergence for different combinations of Force-Fields and water models for the At2g23090.

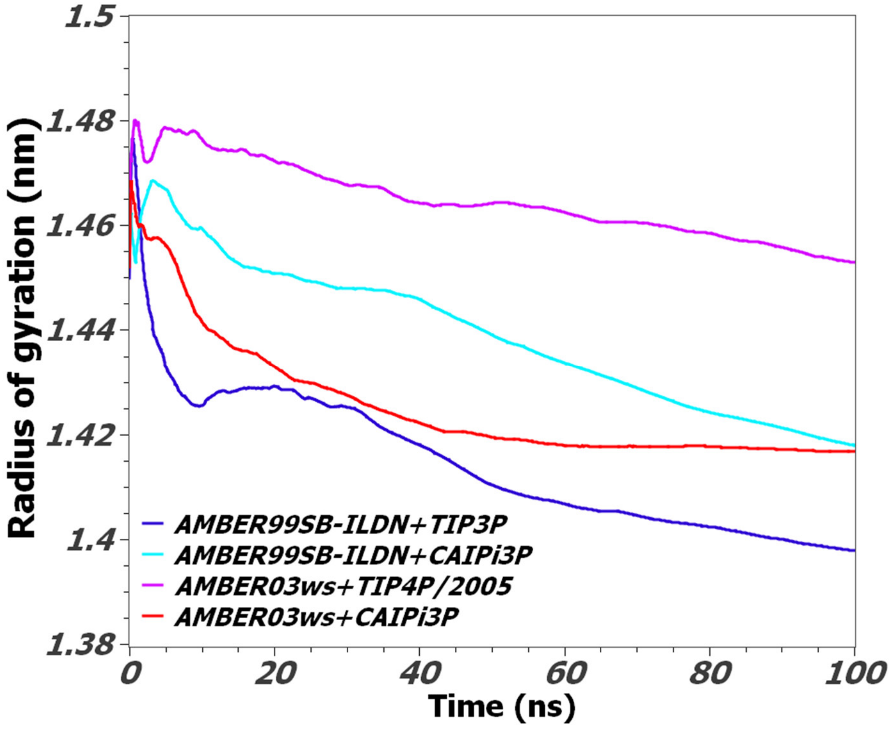

Figure S13. Radius of gyration convergence for different combinations of Force-Fields and water models for the LaRP-RRM1.

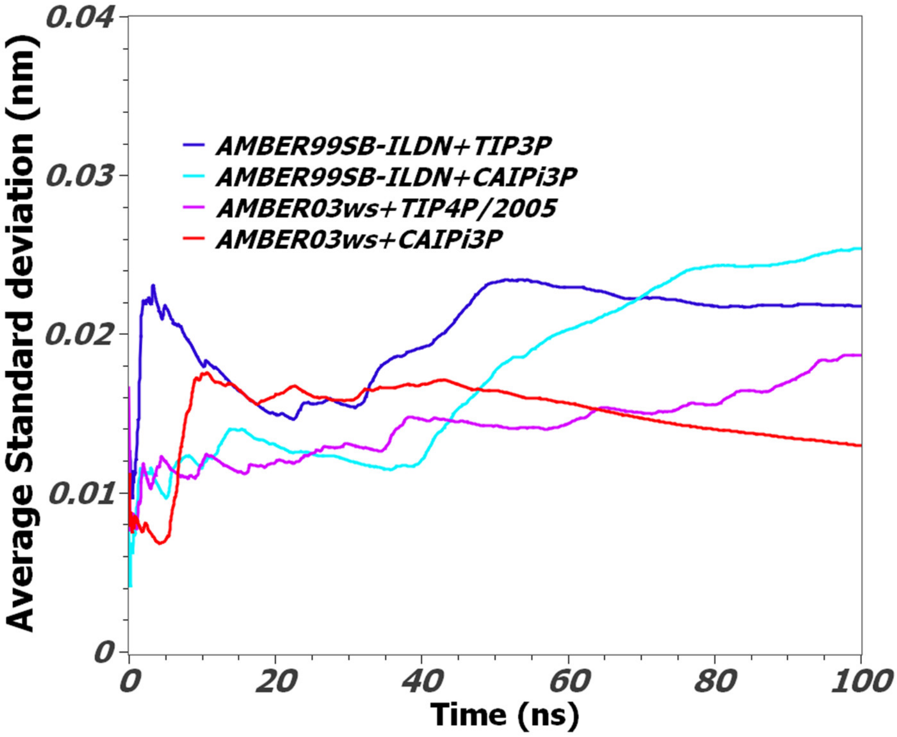

Figure S14. Radius of gyration standard deviation convergence for different combinations of Force-Fields and water models for the LaRP-RRM1.

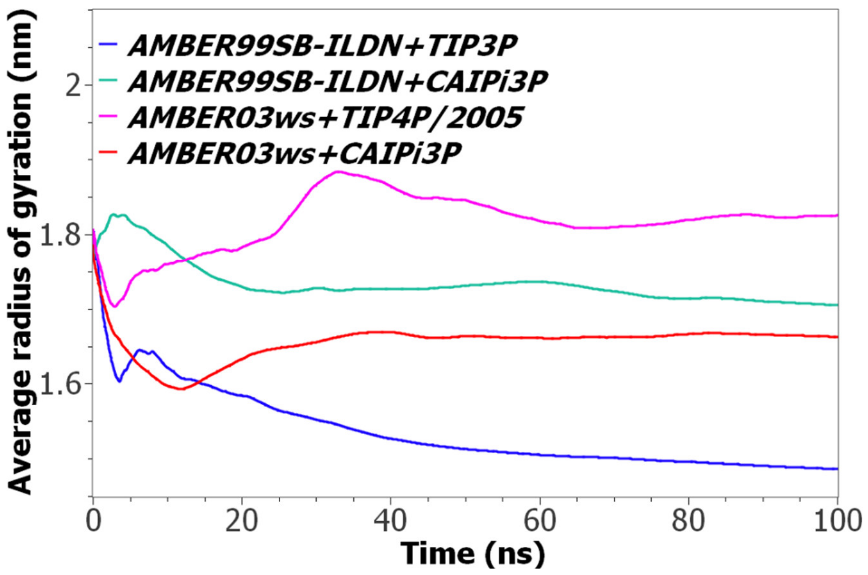

Figure S15. Radius of gyration convergence for different combinations of Force-Fields and water models for the LaRP-RRM1.

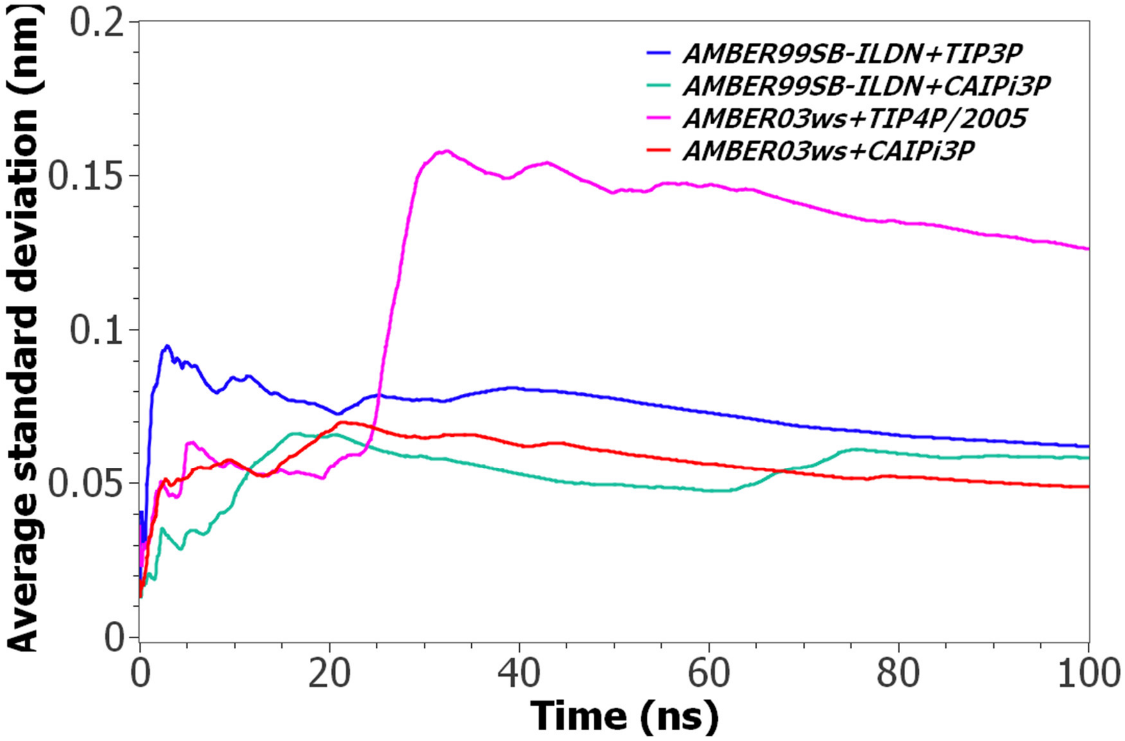

Figure S16. Radius of gyration standard deviation convergence for different combinations of Force-Fields and water models for the LaRP-RRM1.
